# ARTZ @ Jefferson: How Arts-Based Experiences Support People With Dementia as Mentors and Aid in Dignity Preservation

**DOI:** 10.1093/geroni/igaa057.3087

**Published:** 2020-12-16

**Authors:** Susan Shifrin, Florence Gelo, Anne Mitchell

**Affiliations:** 1 ARTZPhilly, Philadelphia, Pennsylvania, United States; 2 Drexel University, Philadelphia, Pennsylvania, United States; 3 Thomas Jefferson University, Philadelphia, Pennsylvania, United States

## Abstract

ARTZ @ Jefferson positions people with dementia and care partners as authorities about their lived experiences; arts-based experiences assist them in communicating with and mentoring health-professions students about those lived experiences. Since Spring 2016, over 100 students have been mentored by people with dementia and their care partners. Their first encounters take place in museum galleries, through facilitated conversations about works of art. Over the next six to eight weeks, students and mentors build relationships through group meetings and individual conversations. Post course surveys demonstrated that nearly 100% of students indicated their increased ability to value listening and listen to others, enhanced the healthcare provider/patient relationship, and prioritized patients’ life experiences. The majority of mentors noted that student interactions added to their quality of life, social engagement and sense of purpose. Preliminary outcomes suggest that arts-based experiences establish mutual respect and empathy between people with dementia and students.

